# Bone Marrow Support of the Heart in Pressure Overload Is Lost with Aging

**DOI:** 10.1371/journal.pone.0015187

**Published:** 2010-12-21

**Authors:** Nikolai A. Sopko, Benjamin A. Turturice, Mitchell E. Becker, Chase R. Brown, Feng Dong, Zoran B. Popović, Marc S. Penn

**Affiliations:** 1 Case Western Reserve University School of Medicine, Cleveland, Ohio, United States of America; 2 Skirball Laboratory for Cardiovascular Cellular Therapeutics, Cleveland Clinic, Cleveland, Ohio, United States of America; 3 Department of Stem Cell Biology and Regenerative Medicine, Cleveland Clinic, Cleveland, Ohio, United States of America; 4 Department of Cardiovascular Medicine, Cleveland Clinic, Cleveland, Ohio, United States of America; Clinica Universidad de Navarra, Spain

## Abstract

**Rationale:**

Exogenous stem cell delivery is under investigation to prevent and treat cardiac dysfunction. It is less studied as to the extent endogenous bone marrow derived stem cells contribute to cardiac homeostais in response to stress and the affects of aging on this stress response.

**Objective:**

To determine the role of bone marrow (BM) derived stem cells on cardiac homeostasis in response to pressure overload (PO) and how this response is altered by aging.

**Methods and Results:**

Young (8 weeks) and old (>40 weeks) C57/b6 mice underwent homo- and heterochronic BM transplantation prior to transverse aortic constriction (TAC). We found that older BM is associated with decreased cardiac function following TAC. This decreased function is associated with decrease in BM cell engraftment, increased myocyte apoptosis, decreased myocyte hypertrophy, increased myocardial fibrosis and decreased cardiac function. Additionally, there is a decrease in activation of resident cells within the heart in response to PO in old mice. Interestingly, these effects are not due to alterations in vascular density or inflammation in response to PO or differences in *ex vivo* stem cell migration between young and old mice.

**Conclusions:**

BM derived stem cells are activated in response to cardiac PO, and the recruitment of BM derived cells are involved in cardiac myocyte hypertrophy and maintenance of function in response to PO which is lost with aging.

## Introduction

The role of adult stem cells in cardiac repair has been the focus of intense research, driven by the goal of developing novel therapies aimed at regenerating damaged myocardium. Several adult stem cell populations have been shown to enhance cardiac repair, including bone marrow (BM) and cardiac based stem and progenitor cell populations, each with potentially different mechanisms and degrees of effect [1], [2], [3]. However, most of the research has focused on their role in acute and chronic myocardial infarction (MI). A smaller number of studies has focused on the involvement of bone marrow stem cells (BMSC) in pressure overload (PO), a clinical condition caused by the increasingly prevalent diseases of aortic stenosis and hypertension [4], [5]. In response to the increased workload and systolic wall stress, the myocardium undergoes hypertrophy and to a lesser extent, hyperplasia, transiently maintaining adequate pump function [5], [6]. However, with time these compensatory mechanisms become inadequate and heart failure ensues [7]. Excessive myocardial hypertrophy, reactive fibrosis, and capillary rarefaction have all been implicated in the transition to failure however their exact mechanisms remain under study [8], [9].

Several groups have identified stem cell involvement in PO in both human and animal studies [4], [6], [10]. Urbanek et al. observed increased numbers of resident cardiac stem cells (CSC) in biopsies from patients undergoing aortic valve replacement. Mueller et al. demonstrated increased endothelial progenitor cell recruitment into the myocardium in mice following transverse aortic constriction (TAC). The impact of aging in this setting is less well addressed despite advanced age being frequently associated with the development of heart failure [4]. Reports have shown the effects of aging on various stem cell compartments in animals and humans. Intrinsic stem cell changes with aging such as increased activity of cell cycle regulatory pathways can lead to decreased stem cell populations and decreased stem cell function [11], [12], [13]. Aging of the stem cell supportive niche or systemic environment can also have negative effects as seen in aged skeletal muscle satellite cells that show improved function when exposed to younger serum [14].

Here we make the novel observation that the BM is involved in supporting the myocardium in chronic PO and that this support is lost with aging. We demonstrate that older BM is associated with decreased cardiac function in the setting of chronic TAC and that this decreased function is associated with increased fibrosis, decreased myocyte hypertrophy, increased apoptosis and decreased BM cell engraftment in the myocardium. Interestingly, these effects of aging are not due to alterations in vascular density or inflammation in response to PO or differences in *ex vivo* stem cell migration between young and old BM. Additionally, we show with age a decrease in activation of resident cells within the myocardium in response to PO. Our findings suggest BM stem cells provide anti-apoptotic, pro-hypertrophic support to myocytes leading to preservation of cardiac function and mitigating adverse cardiac remodeling. These effects are attenuated with age possibly due to a specific BM population of cardioprotective stem cells that may be the origin of cardiac progenitor cells.

## Methods

See Supplemental Material S1 for additional information.

### Animals

All animal experiments were approved by the Cleveland Clinic Institutional Animal Care and Use Committee (ARC-08574). Male C57BL/6J mice (Jackson Laboratories, 000664) were used in all experiments unless otherwise noted. Young mice were aged 8 weeks and old mice were aged 40 weeks at the time of operation. Male C57BL/6J ROSA26 mice expressing beta-galactosidase (Jackson Laboratories, 002192) were used for bone marrow transplantations (BMTx). Male C57BL/6J mice with constitutively active and ubiquitous luciferase expression (Jackson Laboratories, 002709) were used for MSC injections.

### Transverse Aortic Constriction

Transverse aortic constriction was performed as previously described [15]. Briefly, a 7-0 silk suture was constricted around the transverse aorta against an externally positioned 27 gauge needle which was removed after tightening. Age-matched sham animals underwent the same procedure without tightening of the suture. A total of 91 mice survived surgery and 68 (72%) survived until tissue collection, 38 of which were old mice. Numbers of animals per experiment are indicated in their respective legends.

### Bone Marrow Transplantation

Bone marrow transplantations using donor ROSA26 mice were performed as previously described [16]. Irradiated mice received 1 million whole BM cells via femoral injection in both legs. After 5 weeks, mice were assessed for hematologic reconstitution by complete blood count (Drew Scientific Hemavet) and subjected to TAC and sham operations as described above.

### Echocardiographic Assessment of Heart Function

Transthoracic echocardiography was performed by blinded observers as previously described [17]. M-mode images were used to assess cardiac function.

### Histological Analysis

Heart tissue was fixed and embedded in paraffin. Five micron short axis sections from the level of the papillary muscles were cut and underwent Masson's trichrome staining for collagen. Whole sections were imaged at 200× magnification via bright field microscopy (Leica DM5000B). Cardiomyocyte diameter was measured using trans-nuclear width of the shortest diameter of at least 60 myocytes per section as previously described [18] Cardiac fibrosis was assessed by measuring the collagen–stained area as a percentage of total myocardial area as previously described [17].

Macrophages were quantified as previously described [19], [20]. Sections were incubated with a Mac-3 antibody (BD Biosciences). A peroxidase DAB detection system (Vector Laboratories) was used per manufacturer's instructions and sections were counterstained with hematoxylin.

For vessel quantification and myocyte density, sections were incubated with fluorescein-conjugated isolectin (Vector Laboratories), labeling endothelium and rhodamine-conjugated wheat germ agglutinin (Vector Laboratories), which labels myocyte membranes as previously described [21]. Five randomly chosen cross-sectional fields for each sample were imaged at 400× using confocal microscopy (Leica).

Apoptotic nuclei were determined by terminal deoxynucleotidyl transferase-mediated dUTP nick-end labeling (TUNEL) (Roche) staining per manufacturer's instructions, and cardiomyocytes were identified by troponin staining as previously described.

All quantitative evaluations were performed by a blinded observer using ImagePro Plus software (v61.0.346 Media Cybernetics) as previously described [18].

### Bone Marrow Migration

Whole BM was isolated as described above and depleted of lineage positive cells using EasySep Mouse Hematopoietic Progenitor Enrichment Kit (Stem Cell Technologies) depleting CD5, CD11b, CD19, CD45R, Ly-6G, TER119, 7/4 positive cells. 350,000 cells in 0.5% FBS-AMEM in 5 uM pore inserts (Millipore) were placed in a 6-well plate with 0.5% FBS-AMEM media with or without either 1.5 ng/mL or 150 ng/mL recombinant mouse stromal cell-derived factor-1α (SDF-1, R&D Systems) or 3 ng/mL or 300 ng/mL recombinant mouse monocyte chemotactic protein-3 (MCP-3, R&D Systems). Cells were counted with a hemocytometer after migrating for 4 hours at 37°C in hypoxic conditions (5% O_2_, 5% CO_2_).

### Flow Cytometric Analysis

Migrated cells were incubated with PE-, or APC- conjugated monoclonal antibodies against c-Kit and stem cell antigen-1 (SCA-1) (Becton Dickinson). The appropriate conjugated isotype-matched IgGs were used as controls. Cells were analyzed using a LSR II with FACSDiva software (Becton Dickinson).

### Chemokine Gene Expression

RNA was isolated using the guanidinium thiocyanate-phenol-chloroform method (TRIzol, Invitrogen) and reverse transcribed using random hexemer primers (New England BioLabs). Quantitative PCR (qPCR) of cDNA was performed with TaqMan probes (Applied Biosystems) for the genes of interest SDF-1 (Mm00445552_m1) or MCP-3 (Mm00443113_m1). Expression was normalized to endogenous 18s expression (Hs99999901_s1). Data are reported as relative quantification values (RQ) using the ddCt method as previously described. [22].

### Proliferating cell assessment

Twice daily 0.05 mg/g of BrdU (Sigma) was given intraperitoneally for three days prior to collecting hearts 2 weeks post-surgery in young and old non-BMTx mice. Hearts were collected and prepared for immunostaining as previously mentioned. Sections were incubated with a fluorescein conjugated monoclonal antibody to BrdU (Roche), a monoclonal anti-cardiac specific troponin-t antibody (Abcam), wheat germ agglutinin conjugated with Alexa Fluor 647 (Molecular Probes) and DAPI (Vector Laboratories). Incubation with Alexa Fluor 568 goat anti-mouse IgG antibody (Molecular Probes) for troponin-t secondary labeling was performed. Eight random fields per animal at 630x were imaged using confocal microscopy (Leica).

### Detection and Quantification of Bone Marrow Cells in the Heart

Genomic DNA was isolated from left ventricles of BMTx animals using the guanidinium thiocyanate-phenol-chloroform method (TRIzol, Invitrogen). qPCR was performed using genomic DNA and beta-galactosidase primers (for-CCG GAT TGA TGG TAG TGG TC; rev-AAT CCA TCT TGT TCA ATG GCC GAT C). Expression was normalized to 18s gene content (Applied Biosystems, Hs99999901_s1). Data are reported as relative quantification values (RQ).

### Statistics

Values are represented as mean ± SEM. Paired data were evaluated using Student's *t* test. The between-group differences in changes of left ventricular echocardiography parameters at 2 and 4 weeks after surgery were evaluated using repeated-measures two-way analysis of variance. Similarly, histology data obtained at 4 weeks after surgery were compared by a two-way analysis of variance. Survival curves were determined using Kaplan-Meier analysis and compared with the log-rank test. All statistical analysis was done using SPSS 15.0 software (SPSS Inc, Chicago, Il). *P* value <0.05 was considered statistically significant.

## Results

### Old mice have decreased cardiac function and survival in response to PO

**Figure 1:**
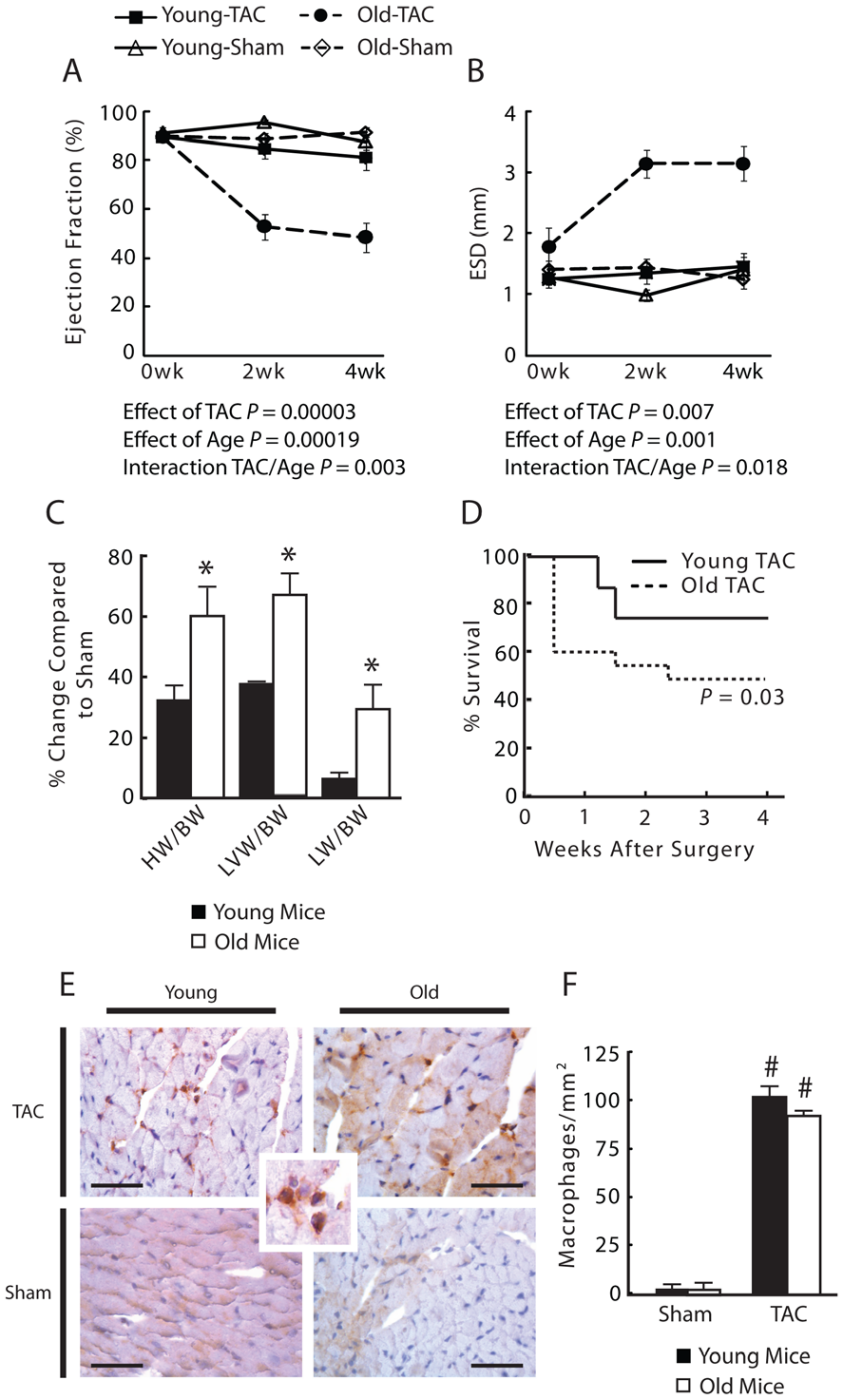
Cardiac function following TAC. (A) Old TAC mice had lower ejection fractions (EF) and (B) greater left ventricular end-systolic dimension (ESD). (C) Old TAC mice had significantly increased heart weight (HW/BW), left ventricle weight (LVW/BW), and lung weight (LW/BW) to body weight ratios compared to young TAC mice 4 weeks after TAC. (D) Old TAC mice had decreased survival compared to young TAC mice at 4 weeks post-TAC. *P<0.05 versus young TAC mice. n = 6–8 per group. (E) Mac-3 staining (example of positive cells in inset) showed increase in macrophage infiltration in TAC animals over sham (#*P*<0.001 compared to shams) but no difference in TAC animals between age groups (*P* = 0.163). n = 3–4 mice per group.

To investigate the effects of age on cardiac compensation in response to chronic PO, we measured cardiac function by echocardiography in young and old mice as a function of time after TAC or sham procedure. Baseline cardiac function parameters showed no difference between young and old mice. However, as expected, left ventricle (LV) mass and body weight were greater in old mice (Table 1). Ejection fraction (EF) was significantly lower in old mice (*P* = 0.00019). Expectedly, TAC reduced EF in young and old mice (*P* = 0.00003, Figure 1B), with the decrease larger in old mice (*P* = 0.003 for TAC/old age interaction). Similarly, TAC significantly increased left ventricular end-systolic dimension (ESD) and was larger in old mice (*P* = 0.007 and *P* = 0.001, respectively, Figure 1B). Again, the TAC-mediated increase was more pronounced in old mice (*P* = 0.018 for TAC/old age interaction). Post-mortem inspection revealed that old TAC animals had significantly increased heart weight (HW/BW), left ventricular weight (LVW/BW), and lung weight (LW/BW) to body weight ratios (Figure 1C) consistent with decreased function. Furthermore, old TAC mice had significantly decreased survival 4 weeks following TAC (*P* = 0.03, Figure 1D). There was no difference in survival rates in sham animals between age groups.

**Table 1:** Young and old mice cardiac parameters at baseline prior to TAC.

	EF (%)	FS (%)	ESD (mm)	EDD (mm)	LV Mass (mg)	Body Weight (g)
Young	89.3±0.05	53.6±0.09	1.3±0.2	2.9±0.2	106.4±26.9	28.3±0.1
Old	89.2±0.07	54.6±0.11	1.4±0.2	3.0±0.2	142.9±9.4	34.3±1.0
*P*-Value	0.99	0.89	0.61	0.99	<0.01	<0.001

Cardiac function does not differ between young and old animals prior to surgery. As expected, old animals have greater LV mass and body weight. EF, ejection fraction; FS, fractional shortening; Mass, left ventricular (LV) mass; ESD, LV end-systolic dimension. n = 6 per group.

### Inflammation following TAC is similar in young and old mice

We examined inflammatory cell infiltration of the left ventricle following TAC between young and old mice as a possible mechanism for the observed differences in cardiac function. Several studies have characterized the inflammatory cell response in pressure overload in young animals [20], [23]. Xia et al. demonstrated no significant neutrophil infiltration following TAC and a peak infiltration of macrophages within 2 weeks which subsided by 28 days. We compared macrophage infiltration 2 weeks following TAC which is when differences in cardiac function between young and old mice became apparent. Both young and old TAC mice had significantly increased macrophage infiltration compared to their respective shams (Figure 1F, 103.6±5.0 vs. 4.2±2.7 cells/mm^2^, *P* = 0.0003 and 93.2±1.6 vs.3.8±3.3 cells/mm^2^, *P* = 0.0004, respectively). However, infiltration between young and old TAC and sham animals did not differ significantly (*P* = 0.16 and *P* = 0.92, respectively).

### Old BM is associated with decreased cardiac function in response to PO

**Figure 2:**
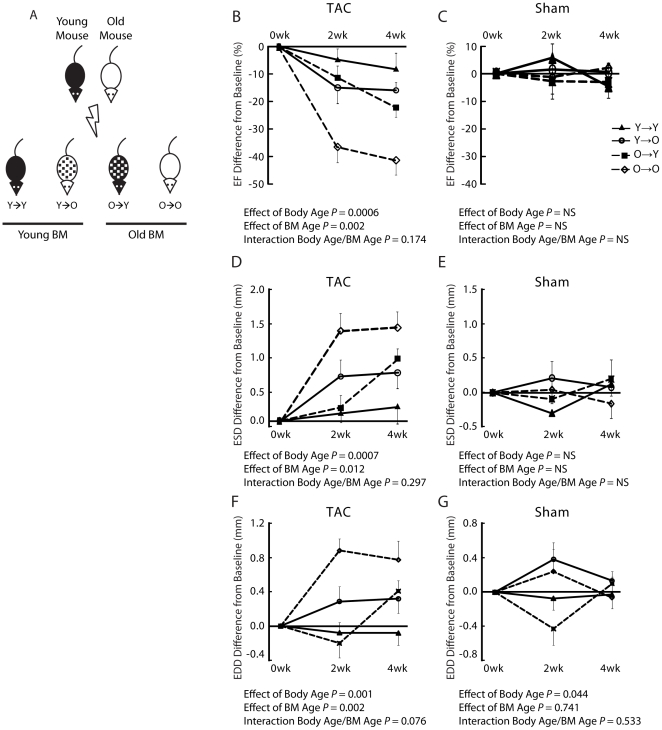
Decreased cardiac function in pressure overload is associated with older bone marrow (BM). (A) Diagram of bone marrow transplantation with β-gal donors. (B, D) Old BM and old body age had significant and independent interactions with cardiac function in TAC mice. There were no differences between Y→O and O→Y groups. (C, E) There were no differences seen between sham animals. n = 7–10 per group. Y→Y, young mice with young BM; Y→O, old mice with young BM; O→Y, young mice with old BM; O→O, old mice with old BM. EF, ejection fraction; EDD, end-diastolic dimension; ESD, end-systolic dimension.

To determine a potential role for BMSC in the myocardial response to PO and whether age attenuates this effect, we performed a series of BMTx between age groups prior to TAC (Figure 2A). Five weeks after BMTx, complete blood counts demonstrated BM reconstitution in all groups (Table S1) and animals underwent TAC or sham operations and were monitored for 4 weeks following surgery. We observed no direct effect of BMTx alone; there were no differences in cardiac function between animals that received age matched BMTx and age matched non-transplanted animals before or after TAC (Figure S1).

At baseline, groups showed no difference in cardiac parameters. BM age and body age had independent additive effects on the response of LV function parameters on TAC. Specifically, EF decreased more in animals with old BM (*P* = 0.002), and in older animals (*P* = 0.0005, Figure 2B), while ESD and EDD increased more in animals with old BM (*P* = 0.012 and *P* = 0.002, respectively), and in older animals (*P* = 0.0006 and *P* = 0.001, respectively) (Figure 2D and 2F), with no interaction between animal age and marrow age (*P* = NS for both interactions). There were no differences in sham cardiac function between groups (Figure 2C, 2E, and 2G).

### Older marrow is associated with more adverse remodeling and less compensatory myocyte hypertrophy in PO

**Figure 3:**
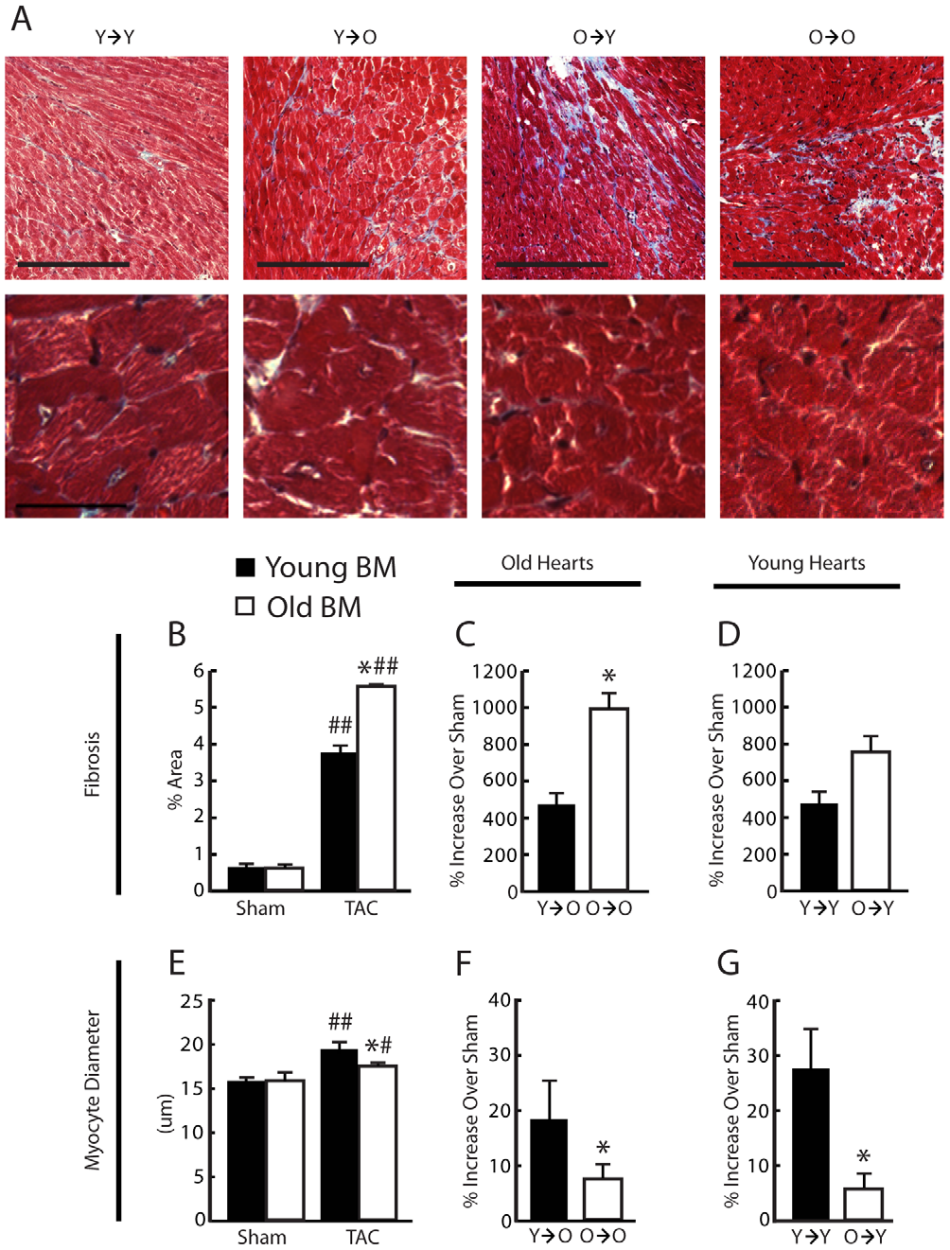
Mice with older bone marrow (BM) have increased cardiac remodeling compared to young BM mice 4-weeks post-TAC. (A) Masons trichrome staining for collagen. (B) Young BM mice had increased myocyte hypertrophy compared to old BM mice. n = 12–14 per group. (C) Young animals with old BM (O→Y) had decreased myocyte hypertrophy compared to young animals with young BM (Y→Y) n = 5–7 per group. (D) Old mice with young BM (Y→O) had increased myocyte hypertrophy compared to old animals with old marrow (O→O). n = 5–7 per group. (E) Old BM mice had increased fibrosis compared to young BM mice. n = 12–14 per group. (F) O→Y mice did not have significantly greater fibrosis than Y→Y mice. n = 5–7 per group. (G) Y→O mice had reduced fibrosis compared to O→O. n = 5–7 per group. #P<0.05 versus respective sham; ##P<0.01 versus respective sham; *P<0.05 versus young BM. Bar represents 200 µM in low magnification and 50 µM in high magnification fields.

To further characterize the effects of BM age in PO, myocardial fibrosis and myocyte hypertrophy were measured. All TAC groups experienced significant increases in fibrosis compared to their respective shams (Figure 3B). As expected, there were no differences in sham animals between groups. In TAC mice, the extent of tissue fibrosis was less in animals with young BM, including old mice with young BM, compared to animals with old BM, including young mice with old BM (3.8±0.3% vs. 5.5±0.6% fibrotic area, *P*<0.05) (Figure 3B). When comparing individual groups, old TAC mice with young BM had less fibrosis than old mice with old BM (462.6±85.8% vs. 974.9±106.1% increase over sham, *P*<0.01) (Figure 3C). Fibrosis in young mice with old BM was not significantly greater than young mice with young BM (Figure 3D).

Similarly, all TAC mice had increased myocyte diameters compared to their respective shams (Figure 3E). There were no differences between sham groups. However, TAC mice with young BM had greater myocyte diameters than mice with old BM (19.5±0.7 µm vs. 17.2±0.5 µm, *P*<0.05) (Figure 3E). When comparing individual groups old mice with young BM had an increased hypertrophic response compared to old animals with old BM (18.5±2.6% vs. 7.4 vs. 3.2% increase over sham, *P*<0.05) (Figure 3F). Young mice with old BM had a reduced hypertrophic response compared to young mice with young BM (6.0±2.2% vs. 27.7±7.1% increase over sham) (Figure 3G).

### Increased myocyte death without changes in vascular density is associated with older BM

**Figure 4:**
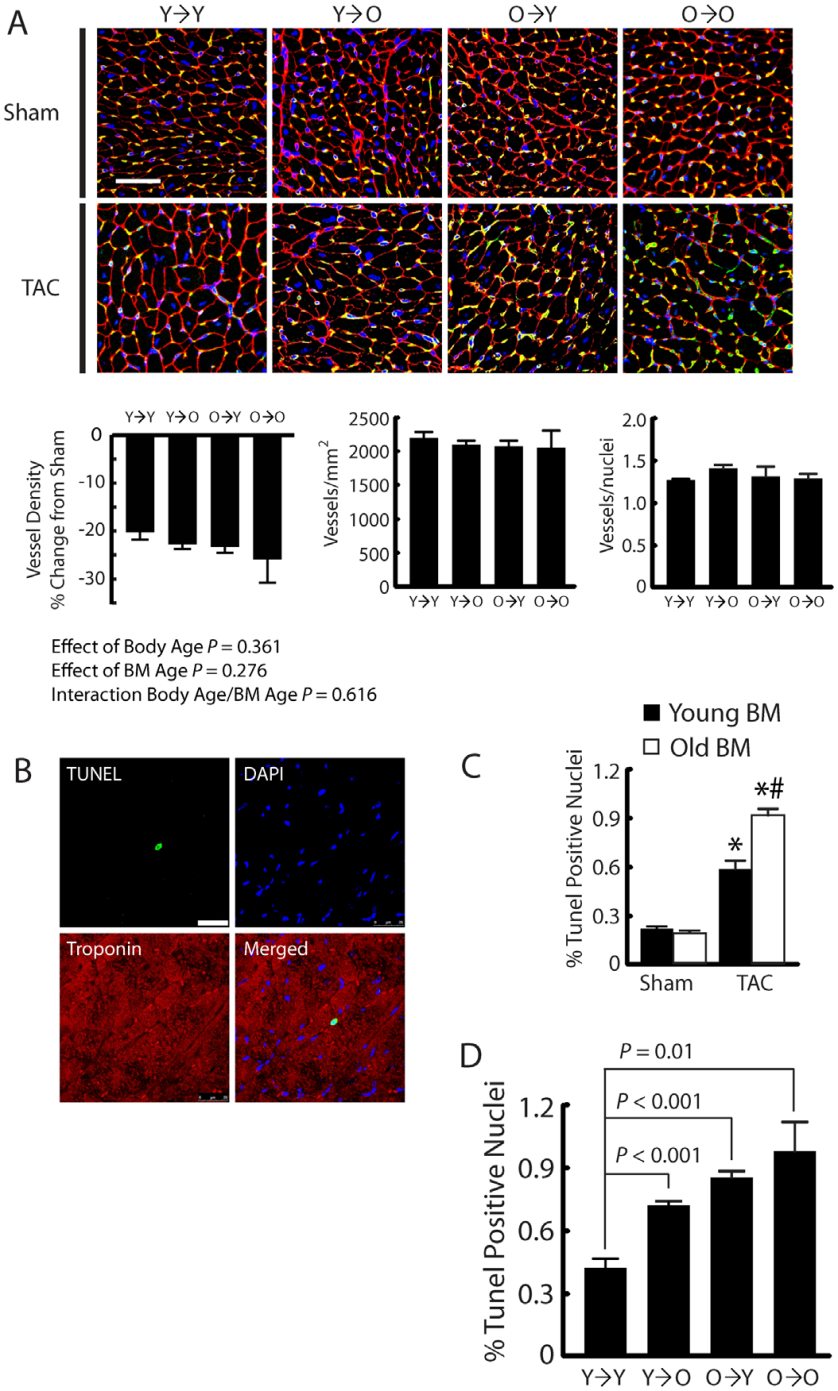
Myocyte death not vessel density is affected by age. (A) Representative pictomicrographs of vascular density from BMTx mice. Vessels are identified by isolectin B4 (yellow) immunohistochemistry. WGA was used to mark cell membranes (red); DAPI identifies nuclei (blue). All TAC groups had significant decreases compared to their respective shams but there was no difference in degree of rarefaction between young and old mice. (B) Representative pictomicrograph of TUNEL positive myocyte. Triple staining (TUNEL, green; DAPI, blue; troponin, red) was performed 4 weeks after TAC. (C) Both young and old bone marrow (BM) TAC animals show significantly increased apoptosis compared to their respective sham (*, *P*<0.001), however old BM mice (O→O, O→Y) have increased apoptosis compared to young BM mice (Y→Y, Y→O) (^#^ *P* = 0.03) n = 7–9 per group. (D) Old BM and old body age are associated with increased TUNEL positive nuclei in TAC mice. n = 5–7 per group. Y→Y, young mice with young BM; Y→O, old mice with young BM; O→Y, young mice with old BM; O→O, old mice with old BM. Bar represents 50 µM.

Myocyte hypertrophy in response to PO has been shown to require vascular growth [8], [24]. We hypothesized that the benefit of young BM could be related to the ability to generate an increase in vascular density and/or minimize vascular rarefaction. To investigate this relationship we assessed vascular density by immunofluorescence in cross-sections of the myocardium 4 weeks following surgery. Although there was evidence of significant vascular rarefaction in all TAC animals, there was no significant difference in the extent of rarefaction between animals transplanted with young and old BM (Figure 4A).

Since rates of vascular rarefaction in response to TAC is not affected by age we next examined myocyte dropout. Increased myocyte apoptosis has been implicated in the transition to heart failure in PO [25], [26]. Increased rates of myocyte loss in animals with old marrow is suggested by the observed increase in collagen deposition which could be in part due to replacement fibrosis following myocyte loss. Therefore we examined myocyte death via TUNEL staining 4 weeks following surgery. All TAC animals demonstrated significant increases in TUNEL staining compared to their respective shams (Figure 4B). Additionally, mice with old BM, including young mice with old BM, had increased TUNEL staining compared to animals with young BM, including old mice with young BM (old vs. young: 0.91±0.07% vs. 0.59±0.06% positive nuclei, *P*<0.01). Comparing individual BMTx groups, effects of BM and body age were seen. All TAC groups had greater TUNEL positive nuclei compared to young mice with young BM (Figure 4D). Both heterochronic groups had similar TUNEL staining results (0.73±0.02% vs. 0.86±0.03% positive nuclei, *P* = 0.388) and were not significantly different than old animals that received old BM (Figure 4D).

### Older age is associated with decreased activation/proliferation of cells and recruitment of BM cells to the myocardium following TAC

**Figure 5:**
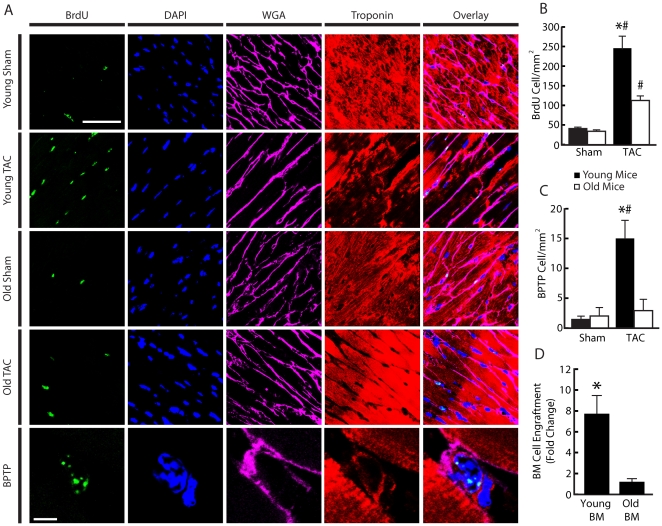
Young animals had increased proliferating cells, cardiac stem cell (CSC) like cells, and bone marrow (BM) derived cells compared to old animals following TAC. (A) Representative staining of proliferating cells and CSC like, small BrdU positive troponin positive (BPTP) cells in young and old non-bone marrow transplanted animals. Quadruple staining (BrdU, green; DAPI, blue; WGA, purple; troponin, red) was performed. (B) Young mice had more proliferating cells in their myocardium compared to old animals 2 weeks post-TAC. (C) Young mice had more CSC like cells compared to old animals 2 weeks post-TAC. (D) Mice with young BM (Y→Y, Y→O) had more cumulative BM derived cells 4-weeks post-TAC than mice with old BM (O→O, O→Y) as measured by PCR for genomic DNA unique to transplanted marrow. ^#^ *P*<0.05 versus respective sham; *P<0.05 versus old mice; n = 5 per group. Bar in upper panel represents 50 µM and 5 µM in lower panel.

Increased proliferation of stem cells in the heart in response to PO has been shown in humans and is associated with cardiac compensation [6]. To investigate the effects of age on cell activation and proliferation in the myocardium following TAC, we quantified cells undergoing DNA synthesis between young and old mice. Non-BMTx animals were pulsed with BrdU for 3 days prior to collection 2 weeks post-TAC. This is when differences in cardiac function between age groups became apparent and when stem cell involvement could be important for preserving cardiac function. The number of BrdU positive cells and CSC like, i.e., small BrdU positive troponin positive cells (BPTP) [6], which were identified by quadruple staining, were measured. Consistent with previous studies, a negligible number (only 3 total) of BrdU positive myocytes were found. Both TAC groups had more BrdU positive cells compared to their respective sham controls, however young TAC mice had more BrdU positive cells than old TAC mice (245.3±30.7 vs 112.2±12.4 BrdU cells/mm^2^, *P*<0.05) (Figure 5B). Importantly, young TAC mice had significantly more BPTP cells compared to their respective sham controls, representing a 10-fold increase. This was not the case in old mice. Furthermore, compared to old TAC, young TAC mice had significantly more BPTP (15.0±3.0 vs. 3.1±1.8 BPTP/mm^2^, *P*<0.05) (Figure 5C).

To assess the effects of age on BM support of the myocardium we compared level of BM derived cells in the left ventricle four weeks following TAC between young BM and old BM animals. We performed qPCR of genomic DNA for the beta-galactosidase gene unique to the transplanted BM that was derived from the ROSA26 mice. Sham animals showed no difference between age groups, however mice with young BM (young and old animals that received young BM) demonstrated a 7-fold increase (*P*<0.05) in BM derived cells in the left ventricle compared to mice with old BM (young and old mice that received old BM) (Figure 5D). Old mice with old marrow demonstrated no increase in BM derived cells compared to sham (0.81±0.40 fold change compared to sham).

### No difference in *ex vivo* migration capacity between young and old BM

**Figure 6:**
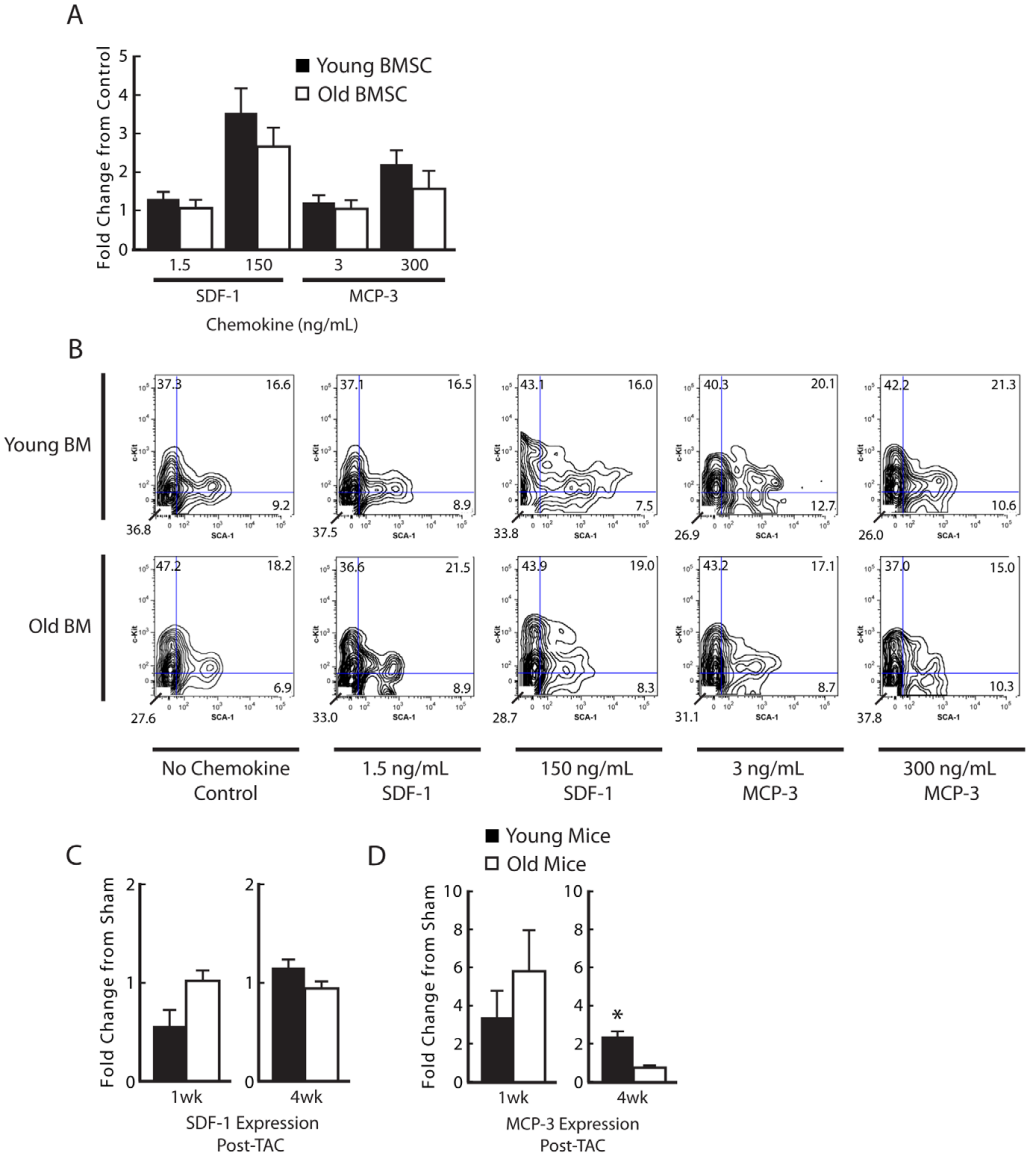
No difference in ex vivo migration potential between young and old bone marrow (BM) progenitor cells. (A) There was no difference in migration between non-manipulated young and old BM progenitor cells exposed to low and high concentrations of SDF-1 and MCP-3. (B) Both young and old non-bone marrow transplanted TAC animals had increased cardiac MCP-3 expression 1-week after TAC, however old TAC mice expression returned to baseline by 4-weeks whereas young TAC mice expression levels remained elevated. (C) There was no difference in SDF-1 expression at 1- or 4-weeks post-TAC between age groups. (D) FACS analysis of c-Kit and SCA-1 positive populations of migrated young and old BMSC showed no difference in bone marrow stem cell population composition. *P<0.05 versus old animals. n = 5 per group.

Based on our observation of decreased stem cell engraftment into the myocardium of older animals, we wanted to determine if old BMSC lost their migratory capacity or if older myocardium lacked the ability to up regulate stem cell homing factors. The effect of BM transplantation on cardiac function suggests that cells from the BM migrate to the heart in response to PO. Since aging affects the expression of genes involved in transmembrane, G-protein-coupled receptor, and protein tyrosine phosphatase signaling activity [27], we measured the migration potential of lineage depleted young and old BM cells in response to MCP-3 and SDF-1; these two chemokines have been shown to be highly relevant to the recruitment of stem cells to the heart following injury [28], [29]. We compared migration of lineage depleted whole BM from young and old animals to increasing concentrations of SDF-1 and MCP-3. Interestingly, there were no differences in the migratory capacity of primitive cells between young and old BM (Figure 6A). Additionally, FACS analysis failed to detect significant changes in the c-Kit and SCA-1 fractions of migrated cells (Figure 6B). The expression of SDF-1 at 1 and 4 weeks after TAC was not changed in the LV of young and old mice (Figure 6C) whereas both groups demonstrated an increase in MCP-3 expression at 1 week post-TAC compared to their respective sham groups. Interestingly, there was no difference in the magnitude of increase between age groups (young, 3.3±1.5 vs. old, 5.8±2.1, *P* = 0.58) (Figure 6C). However, at 4 weeks after TAC, there was increased expression of MCP-3 in young but not old LV (2.3±0.3 vs. 0.7±0.04, *P*<0.05) (Figure 6D).

## Discussion

In this study, we show that old mice have a worse response to chronic PO, as evidenced by larger end-systolic dimensions, lower ejection fraction, and finally, lower survival. Furthermore, this worse response of older animals is not due to differences in inflammatory cell infiltration but mediated not only by aging of the heart, but also by aging of the BM. It is important to note that the older animals in out studies are middle-aged mice and that the analyses performed could only able be performed on survivors. Older animals had a greater mortality rate in the first week after TAC; therefore, they did not survive long enough to allow for meaningful analyses. Importantly, body age and bone marrow age appear additive and of similar magnitude, as evidenced by a similar decline of cardiac function in young animals transplanted with old BM and old animals transplanted with young BM.

It appears that the effect of young BM is mediated by the suppression of collagen synthesis, stimulation of myocyte hypertrophy, and reduction in myocyte dropout. Consistent with the findings of decreased myocyte density, we further demonstrate that aging of the BM leads to a loss of bone marrow-derived cell engraftment in the myocardium, and a decrease in cardiac progenitor cell activation/proliferation/migration within the myocardial tissue in response to PO.

Over the last decade, a variety of BM cells has been utilized for cardiac repair including hematopoietic stem cells, mesenchymal stem cells and endothelial progenitor cells [30] as well as more specific cardiac based stem cell populations [31], [32], [33]. Comparatively fewer studies have defined the role of endogenous BMSC and aging on trophic support and repair of the heart in response to stress [10], [32]. This report provides several important insights into the mechanisms of BM support of the myocardium in PO and how they are affected with age.

### BMSC based support decreases adverse cardiac remodeling and aids in myocyte survival

Following activation and recruitment of stem cells to the myocardium, which is attenuated with age, stem cells provide trophic support through as of yet unknown mechanism. Paracrine mediators and their effects have been studied extensively in the setting of MI where stem cells can have a significant impact on scar remodeling and myocyte survival [34], [35]. In our heterochronic BMTx model, increased myocyte hypertrophy and decreased fibrosis is associated with young BM.

In addition to hypertrophy and decreased fibrosis, young BMSC are associated with myocyte survival, similar to responses seen in MI. In our model, myocyte death is influenced by age. Young mice with young marrow had decreased myocyte death at 4 weeks compared to old animals with old marrow. Young mice also did not demonstrate a decrease in density whereas animals with old BM did. The increased fibrosis seen with aged BM is likely due in part to reactive scarring in response to myocyte dropout. Old animals demonstrated significantly worse survival compared to young mice and analysis were performed only on animals which survived to tissue collection, thus above differences could be more pronounced.

Surprisingly, there was no difference in degree of vessel rarefaction following TAC between young and old BM suggesting that the benefit associated with young BM in PO is not due to the improved release of endothelial progenitor cells as has been seen in other disease models involving stem cell aging [36]. This does not exclude a role for the BM in angiogenesis in PO which has previously been indicated [37]; however, it appears that this response to TAC may be minimally affected by age.

### Decreased proliferation, activation, and recruitment of stem cells to the heart in response to TAC with age

Both young and old TAC animals had increased proliferating small non-myocyte cells which are most likely a combination of BM and cardiac based stem cells. However, older TAC mice had significantly less. More importantly, older TAC animals did not have an increase in the number of small BrdU positive CSC like cells whereas young TAC animals experienced a 10-fold increase over sham. These data are corroborated by the observation of decreased myocardial recruitment of BM cells after 4 weeks of banding in old BM animals.

Interestingly, *ex vivo* measurements of migration potential showed no difference between young and old BMSC or a difference in stem cell composition of migrated populations, suggesting that at baseline old BM migratory response is comparable to young BM. However, in the chronic state of PO, these effector stem cell populations are exhausted by the second week as suggested by the decline in cardiac function, BrdU proliferation and bone marrow engraftment data.

While the focus of our findings is on the role of BM age on the myocardial response to PO, our study also demonstrates the importance of heart age, which is supported by a large body of important work [38], [39], [40]. Young BM did not completely rescue older TAC animals nor did young TAC animals have as poor of function when given old BM. This could be due to various reasons including altered myocyte responses at the molecular level that are heavily engaged during PO [38], [41]. Decreased myocardial stem cell chemokine expression with age could also contribute to poor function. Older TAC hearts had baseline levels of MCP-3 expression at 4 weeks, which was significantly less than young TAC heart expression. However, young BM was still able to respond to older TAC hearts. It is also possible that CSC populations are affected by age. In our study, old TAC animals had a decrease in BrdU positive and in CSC like BrdU positive troponin positive cells.

Other researchers have shown that the BM acts as a progenitor cell reserve for other non-hematological adult progenitor cell populations [42]. Thus, in a stressed state, CSC are actively involved in repair and their reserves are replaced by BM derived cells. Our findings support this concept of BM replenishment of CSC occurs in PO.

Our data have important implications for understanding the role of the BM in the homeostasis of cardiac function in the setting of PO. We demonstrated that BM derived cells can support the myocardium on a relatively acute basis (<1 month) independent of myocardial regeneration and that this ability is lost with age possibly due to a loss of a specific BM stem cell population. Further defining this population and understanding the role of age in its decline could facilitate our understanding of the role of the bone marrow in maintaining myocardial homeostasis in heart disease as well as the development of novel stem cell targeted adjunct therapies for patients undergoing aortic valve replacement and who have poor cardiac function.

## Supporting Information

Material S1 Detailed methods and data analyses.
(DOC)

Table S1 Complete blood cell counts of bone marrow transplanted animals demonstrate reconstitution 5 weeks following transplantation as compared to young and old animals. n = 5 per group.
(DOC)

Figure S1 Bone marrow transplantation does not affect cardiac function at baseline prior to or after TAC in young mice bone marrow transplanted with young bone marrow (A) or old mice transplanted with old marrow (B) compared to their non-bone marrow transplanted surgical controls. n = 4-6 per group.
(DOC)
