# Supplementary material for: Bone Marrow Support of the Heart in Pressure Overload Is Lost with Aging
Source: PLoS One. 2010 Dec 21;5(12):e15187. doi: 10.1371/journal.pone.0015187 (PMC3006343; doi:10.1371/journal.pone.0015187)
Supplement: Material S1 — Detailed methods and data analyses. (DOC) [file pone.0015187.s001.doc]

**SUPPLEMENTAL MATERIAL**

**METHODS**

**Animals**

Male C57/b6 mice (Jackson Laboratories, 000664) were used in all experiments unless otherwise noted. Young mice were aged 8 weeks and old mice were aged 40 weeks at the time of operation. Male C57/b6 ROSA26 mice expressing beta-galactosidase (Jackson Laboratories, 002192) were used for bone marrow transplantations (BMTx) with donors for young BMTx aged 4 weeks and old BM donors aged 40 weeks. All animal experiments were approved by the Cleveland Clinic Institutional Animal Care and Use Committee.

**Transverse Aortic Constriction**

Transverse aortic constriction was performed as previously described [1]. Briefly animals were anesthetized by intraperitoneal injection of a xylazine/ketamine mixture, ventilated (Harvard apparatus) and restrained in a supine position. Under a dissecting microscope (Leica), a sternotomy was performed, and the transverse aorta between the right subclavian and left common carotid arteries was isolated. A 7-0 silk suture was placed around the transverse aorta and constricted against a 27 gauge needle placed externally on top of the aorta which was removed after tightening leaving the aorta approximately 75% occluded. Once banded, the sternum and skin were approximated in layers and any residual pneumothorax was reduced using a 1cc syringe with a 20 gauge needle. Pulsed wave Doppler echocardiography performed 24h after surgery showed that a pressure gradient across constriction in all animals was at least 64 mmHg. In control age-matched mice, a sham operation without aorta occlusion was performed.

**Bone Marrow Transplantation**

Donor ROSA26 mice were sacrificed and the hind limbs were removed. The femurs and tibias were cut from both ends and the BM was flushed into a 50 ml falcon with flush medium (Alpha Medium with 2 g/L NaHCO_3_, 10% horse serum, 10% FBS, 1% L-Glutamine, 1% penicillin-streptomycin). After collection of BM from femur and tibia, red blood cells were lysed by red blood cell lysis buffer (155 mM NH4Cl, 12 mM NaHCO3, 0.1 mM EDTA). Recipient C57/BL6J mice were pretreated with acidified water (pH = 2-3) and neomycin 1.1 g/liter of water and polymixin 1 million unit/liter of water for 7-10 days. After pretreatment, the mice underwent irradiation at a total dose of 850 rads divided into 2 equal doses of 425 rads separated by 4 hours. The irradiated mice received 1 million whole BM cells via femoral injection in both legs. After 5 weeks, the BM transplanted mice were assessed for hematologic reconstitution by complete blood count (Drew Scientific Hemavet) and were subjected to TAC and sham operations as described above.

**Echocardiographic Assessment of Heart Function**

Transthoracic echocardiography (echo) was performed using a Vivid 7 ultrasound machine (GE Medical) equipped an il3L linear probe operated at 14 MHz. Mice were imaged in a conscious state at a room temperature of 73 °F and with decreased ambient lighting while held by an experienced handler in a supine position. All mice underwent at least one prior echocardiography study in order to become accustomed to handling and the procedure.

The heart was imaged in the 2-dimensional mode in the parasternal long and short-axis views with a depth setting of 1.0 cm and at a frame rate of ≥275 fps. An M-mode image was obtained at a sweep speed of 200 mm/s. Diastolic and systolic LV wall thickness, LV end diastolic dimensions (LVEDD), and LV end-systolic chamber dimensions (LVESD) were measured. All measurements were done from leading edge to leading edge according to the American Society of Echocardiography guidelines [2]. Ejection fraction was calculated using the cubed method. LV mass was calculated using the equation LV mass (mg) =1.055 [(LVEDD+ PWTD+ VSTD) ^3^ − (LVEDD) ^3^], where 1.055 is the gravity of myocardium, PWTD is diastolic posterior wall thickness, and VSTD is diastolic ventricular septum thickness [3].

**Histological Analysis**

Mice were euthanized (KCl 2 mEq IV to arrest the heart in diastole) under deep anesthesia. The hearts were flushed and removed, left ventricles were dissected, and after measuring LV weight, fixed in 10% neutral buffered formalin and embedded in paraffin. Five micron short axis sections were cut and Masson’s trichrome staining for collagen was performed. Whole sections were imaged at 200x magnification via bright field microscopy (Leica DM5000B). Cardiomyocyte diameter was measured using trans-nuclear width of the shortest diameter of at least 60 myocytes per section as previously described [4] Cardiac fibrosis was assessed by measuring the collagen–stained area as a percentage of total myocardial area as previously described [5].

Macrophages were quantified as previously described [6,7]. Sections were incubated with a Mac-3 antibody (BD Biosciences). A peroxidase DAB detection system (Vector Laboratories) was used per manufacturer’s instructions and sections were counterstained with hematoxylin.

For vessel quantification and myocyte density, sections were stained with fluorescein-conjugated isolectin (Vector Laboratories), which stains endothelium and rhodamine-conjugated wheat germ agglutinin (Vector Laboratories), which labels myocyte membranes as previously described [8]. Five randomly chosen cross-sectional fields for each sample were imaged at 400x using confocal microscopy (Leica). All quantitative evaluations were performed with ImagePro Plus software (v61.0.346 Media Cybernetics) [4].

**Bone Marrow Migration**

Whole marrow was isolated as described above and depleted of lineage positive cells using EasySep Mouse Hematopoietic Progenitor Enrichment Kit (Stem Cell Technologies) depleting CD5, CD11b, CD19, CD45R, Ly-6G, TER119, 7/4 positive cells via magnetic separation. 350,000 cells in 0.5% FBS-AMEM in 5uM pore inserts (Millipore) were placed in a 6-well plate with 0.5% FBS-AMEM media with or without either 1.5ng/mL or 150ng/mL recombinant mouse stromal derived factor-1 (SDF-1, R&D Systems) or 3ng/mL or 300ng/mL recombinant mouse monocyte chemoattractant protein-3 (MCP-3, R&D Systems). Cells were counted with a hemocytometer after migrating for 4 hours at 37^o^ C in hypoxic conditions.

**Flow Cytometric Analysis**

Migrated cells were incubated with PE-, or APC- conjugated monoclonal antibodies against c-Kit and stem cell antigen-1 (SCA-1) (Becton Dickinson). The appropriate conjugated isotype-matched IgGs were used as controls. Cells were analyzed using a LSR II with FACSDiva software (Becton Dickinson).

**Chemokine Gene Expression**

Quantitative real-time polymerase chain reaction (qPCR) was performed using the ABI Prism 7500 sequence detector (Applied Biosystems) following isolation of RNA from left ventricular tissue using the Guanidinium thiocyanate-phenol-chloroform method (TRIzol, Invitrogen) and reverse transcribed using random hexemer primers (New England BioLabs). TaqMan Gene Expression Master Mix (Applied Biosystems), with 40ng of cDNA per reaction and TaqMan probes (Applied Biosystems) for the genes of interest SDF-1 (Mm00445552_m1) or MCP-3 (Mm00443113_m1) were used. The reaction was carried out 40 cycles with each cycle consisting of 15 seconds at 95°C followed by 1 minute at 60°C. Results for each animal were normalized to endogenous 18s expression (Hs99999901_s1). Data are reported as relative quantification values (RQ) using the ddCt method as previously described. [9].

**Proliferating cell assessment**

0.05mg/g of 5-Bromodeoxyuridine-2’-deoxyuridine (BrdU, Sigma) was given twice daily for three days prior to collecting hearts 2 weeks post-surgery in young and old mice. Hearts were collected and prepared for immunostaining as previously mentioned. Sections were incubated with a fluorescein conjugated monoclonal antibody to BrdU (Roche), a monoclonal anti-cardiac specific troponin-t antibody (Abcam), wheat germ agglutinin conjugated with Alexa Fluor 647 (Molecular Probes) and 4’,6’-diamidino-2-phenylindole (DAPI, Vector Laboratories). Incubation with Alexa Fluor 568 goat anti-mouse IgG antibody (Molecular Probes) for troponin secondary labeling was performed. Eight random fields per animal at 630x were imaged using confocal microscopy (Leica).

**Mesenchymal Stem Cell Injections**

Bone marrow mesenchymal stem cells (MSCs) were isolated as previously described [10,11] from a young C57/b6 mouse with ubiquitous luciferase expression (Jackson Laboratories, 002709). Briefly, whole BM was collected from young mice as described above. Cells were allowed to adhere and passaged in high serum media. At passage 6 any remaining CD45 and TER119 positive cells were removed by magnetic separation (Stem Cell Technologies). Old animals underwent TAC or sham surgeries as described above. At 1 week following surgery, animals underwent echocardiography as described above to ensure proper TAC and were randomized into saline and MSC injection groups. 100,000 MSCs in 100 uL PBS were injected via the tail vein every other day for a total of 3 injection and 300,000 cells beginning 1 week post-surgery. Saline control animals underwent the same procedure with a 100 uL PBS infused without MSC. Animals were followed by echocardiography weekly for 4 weeks post-surgery (2 weeks after final cell infusion).

**Detection and Quantification of Bone Marrow Cells in the Heart**

To determine BM derived cells were recruited to and engrafted into the heart 4 weeks after TAC genomic DNA was isolated from left ventricle using the Guanidinium thiocyanate-phenol-chloroform (TRIzol, Invitrogen) of those animals that underwent BMTx with ROSA26 bone marrow. Polymerase chain reaction (PCR) for 40 cycles was performed using primers and conditions provided by Jackson Laboratories for the luciferase gene (for-GCGGAATACTTCGAAATGTCC, rev- CCTTAGGTAACCCAGTAGATCC) and an internal control from the t-cell receptor coding region (for-CAAATGTTGCTTGTCTGGTG, rev- GTCAGTCGAGTGCACAGTTT).

Comparative amounts of bone marrow cells in the heart four weeks after TAC of BM transplanted animals were measured by qPCR. Genomic DNA isolated as described above from left ventricles along with beta-galactosidase primers provided by Jackson Laboratories (for-CCG GAT TGA TGG TAG TGG TC; rev-AAT CCA TCT TGT TCA ATG GCC GAT C) were used with SYBR Green Master Mix (Applied Biosystems). Reactions were carried out for 40 cycles with each cycle consisting of 15 seconds at 95°C followed by 1 minute at 60°C using ABI Prism 7500 sequence detector (Applied Biosystems). Results for each animal were normalized to endogenous 18s expression (Hs99999901_s1). Data are reported as relative quantification values (RQ) using the ddCt method.

**Statistics**

Values are represented as mean ± SEM. Paired data were evaluated using Student’s *t* test. The between-group differences in changes of left ventricular echocardiography parameters at 2 and 4 weeks after surgery were evaluated using repeated-measures two-way analysis of variance. Similarly, histology data obtained at 4 weeks after surgery were compared by a two-way analysis of variance. Survival curves were determined using Kaplan-Meier analysis and compared with the log-rank test. All statistical analysis was done using SPSS 15.0 software (SPSS Inc, Chicago, Il). *P* value <0.05 was considered statistically significant.

**SUPPLEMENTAL REFERENCES**

1. Rockman HA, Ross RS, Harris AN, Knowlton KU, Steinhelper ME, et al. (1991) Segregation of atrial-specific and inducible expression of an atrial natriuretic factor transgene in an in vivo murine model of cardiac hypertrophy. Proc Natl Acad Sci U S A 88: 8277-8281.

2. Lang RM, Bierig M, Devereux RB, Flachskampf FA, Foster E, et al. (2005) Recommendations for chamber quantification: a report from the American Society of Echocardiography's Guidelines and Standards Committee and the Chamber Quantification Writing Group, developed in conjunction with the European Association of Echocardiography, a branch of the European Society of Cardiology. J Am Soc Echocardiogr 18: 1440-1463.

3. Devereux RB, Alonso DR, Lutas EM, Gottlieb GJ, Campo E, et al. (1986) Echocardiographic assessment of left ventricular hypertrophy: comparison to necropsy findings. Am J Cardiol 57: 450-458.

4. Bian J, Popovic ZB, Benejam C, Kiedrowski M, Rodriguez LL, et al. (2007) Effect of cell-based intercellular delivery of transcription factor GATA4 on ischemic cardiomyopathy. Circulation research 100: 1626-1633.

5. Peng Y, Popovic ZB, Sopko N, Drinko J, Zhang Z, et al. (2009) Speckle tracking echocardiography in the assessment of mouse models of cardiac dysfunction. Am J Physiol Heart Circ Physiol 297: H811-820.

6. Gong Y, Hart E, Shchurin A, Hoover-Plow J (2008) Inflammatory macrophage migration requires MMP-9 activation by plasminogen in mice. J Clin Invest 118: 3012-3024.

7. Xia Y, Lee K, Li N, Corbett D, Mendoza L, et al. (2009) Characterization of the inflammatory and fibrotic response in a mouse model of cardiac pressure overload. Histochem Cell Biol 131: 471-481.

8. Hilfiker-Kleiner D, Hilfiker A, Kaminski K, Schaefer A, Park JK, et al. (2005) Lack of JunD promotes pressure overload-induced apoptosis, hypertrophic growth, and angiogenesis in the heart. Circulation 112: 1470-1477.

9. Livak KJ, Schmittgen TD (2001) Analysis of relative gene expression data using real-time quantitative PCR and the 2(-Delta Delta C(T)) Method. Methods 25: 402-408.

10. Schenk S, Mal N, Finan A, Zhang M, Kiedrowski M, et al. (2007) Monocyte chemotactic protein-3 is a myocardial mesenchymal stem cell homing factor. Stem cells (Dayton, Ohio) 25: 245-251.

11. Wieczorek G, Steinhoff C, Schulz R, Scheller M, Vingron M, et al. (2003) Gene expression profile of mouse bone marrow stromal cells determined by cDNA microarray analysis. Cell Tissue Res 311: 227-237.
