# Supplementary material for: Bone Marrow Support of the Heart in Pressure Overload Is Lost with Aging
Source: PLoS One. 2010 Dec 21;5(12):e15187. doi: 10.1371/journal.pone.0015187 (PMC3006343; doi:10.1371/journal.pone.0015187)
Supplement: Table S1 — Complete blood cell counts of bone marrow transplanted animals demonstrate reconstitution 5 weeks following transplantation as compared to young and old animals. n = 5 per group. (DOC) [file pone.0015187.s002.doc]

**Table S**1

|  | **Units** | **Range** | **Young** | **Y→O** | **O→Y** | **Old** |
| --- | --- | --- | --- | --- | --- | --- |
| **WBC** | K/uL | 1.8 - 10.7 | 9.24 | 6.61 | 6.27 | 14.74 |
| **NE** | K/uL | 0.1 - 2.4 | 0.31 | 1.82 | 2.08 | 0.45 |
| **LY** | K/uL | 0.9 - 9.3 | 8.46 | 4.36 | 3.58 | 13.72 |
| **MO** | K/uL | 0.0 - 0.4 | 0.14 | 0.34 | 0.30 | 0.23 |
| **EO** | K/uL | 0.0 - 0.2 | 0.26 | 0.08 | 0.24 | 0.29 |
| **BA** | K/uL | 0.0 - 0.2 | 0.05 | 0.02 | 0.07 | 0.02 |
| **RBC** | M/uL | 6.36 - 9.42 | 7.9 | 8.65 | 6.50 | 7.34 |
| **Hb** | g/dL | 11.0 - 15.1 | 11.76 | 11.09 | 10.40 | 9.85 |
| **HCT** | % | 35.1 - 45.4 | 44.0 | 37.13 | 30.74 | 36.83 |
| **PLT** | K/uL | 592 - 2972 | 955.20 | 568.6 | 423.6 | 1538.96 |
|  |  |  |  |  |  |  |

WBC, white blood cell; NE, neutrophil; LY, lymphocyte; MO, monocyte; EO, eosinophil; BA, basophil; RBC, red blood cell; Hb, hemoglobin; HCT, hematocrit; PLT, platelet

**SUPPLEMENTAL REFERENCES**

1. Rockman HA, Ross RS, Harris AN, Knowlton KU, Steinhelper ME, et al. (1991) Segregation of atrial-specific and inducible expression of an atrial natriuretic factor transgene in an in vivo murine model of cardiac hypertrophy. Proc Natl Acad Sci U S A 88: 8277-8281.

2. Lang RM, Bierig M, Devereux RB, Flachskampf FA, Foster E, et al. (2005) Recommendations for chamber quantification: a report from the American Society of Echocardiography's Guidelines and Standards Committee and the Chamber Quantification Writing Group, developed in conjunction with the European Association of Echocardiography, a branch of the European Society of Cardiology. J Am Soc Echocardiogr 18: 1440-1463.

3. Devereux RB, Alonso DR, Lutas EM, Gottlieb GJ, Campo E, et al. (1986) Echocardiographic assessment of left ventricular hypertrophy: comparison to necropsy findings. Am J Cardiol 57: 450-458.

4. Bian J, Popovic ZB, Benejam C, Kiedrowski M, Rodriguez LL, et al. (2007) Effect of cell-based intercellular delivery of transcription factor GATA4 on ischemic cardiomyopathy. Circulation research 100: 1626-1633.

5. Peng Y, Popovic ZB, Sopko N, Drinko J, Zhang Z, et al. (2009) Speckle tracking echocardiography in the assessment of mouse models of cardiac dysfunction. Am J Physiol Heart Circ Physiol 297: H811-820.

6. Gong Y, Hart E, Shchurin A, Hoover-Plow J (2008) Inflammatory macrophage migration requires MMP-9 activation by plasminogen in mice. J Clin Invest 118: 3012-3024.

7. Xia Y, Lee K, Li N, Corbett D, Mendoza L, et al. (2009) Characterization of the inflammatory and fibrotic response in a mouse model of cardiac pressure overload. Histochem Cell Biol 131: 471-481.

8. Hilfiker-Kleiner D, Hilfiker A, Kaminski K, Schaefer A, Park JK, et al. (2005) Lack of JunD promotes pressure overload-induced apoptosis, hypertrophic growth, and angiogenesis in the heart. Circulation 112: 1470-1477.

9. Livak KJ, Schmittgen TD (2001) Analysis of relative gene expression data using real-time quantitative PCR and the 2(-Delta Delta C(T)) Method. Methods 25: 402-408.

10. Schenk S, Mal N, Finan A, Zhang M, Kiedrowski M, et al. (2007) Monocyte chemotactic protein-3 is a myocardial mesenchymal stem cell homing factor. Stem cells (Dayton, Ohio) 25: 245-251.

11. Wieczorek G, Steinhoff C, Schulz R, Scheller M, Vingron M, et al. (2003) Gene expression profile of mouse bone marrow stromal cells determined by cDNA microarray analysis. Cell Tissue Res 311: 227-237.
