# Supplementary figures and images for: Bone Marrow Support of the Heart in Pressure Overload Is Lost with Aging
Source: PLoS One. 2010 Dec 21;5(12):e15187. doi: 10.1371/journal.pone.0015187 (PMC3006343; doi:10.1371/journal.pone.0015187)

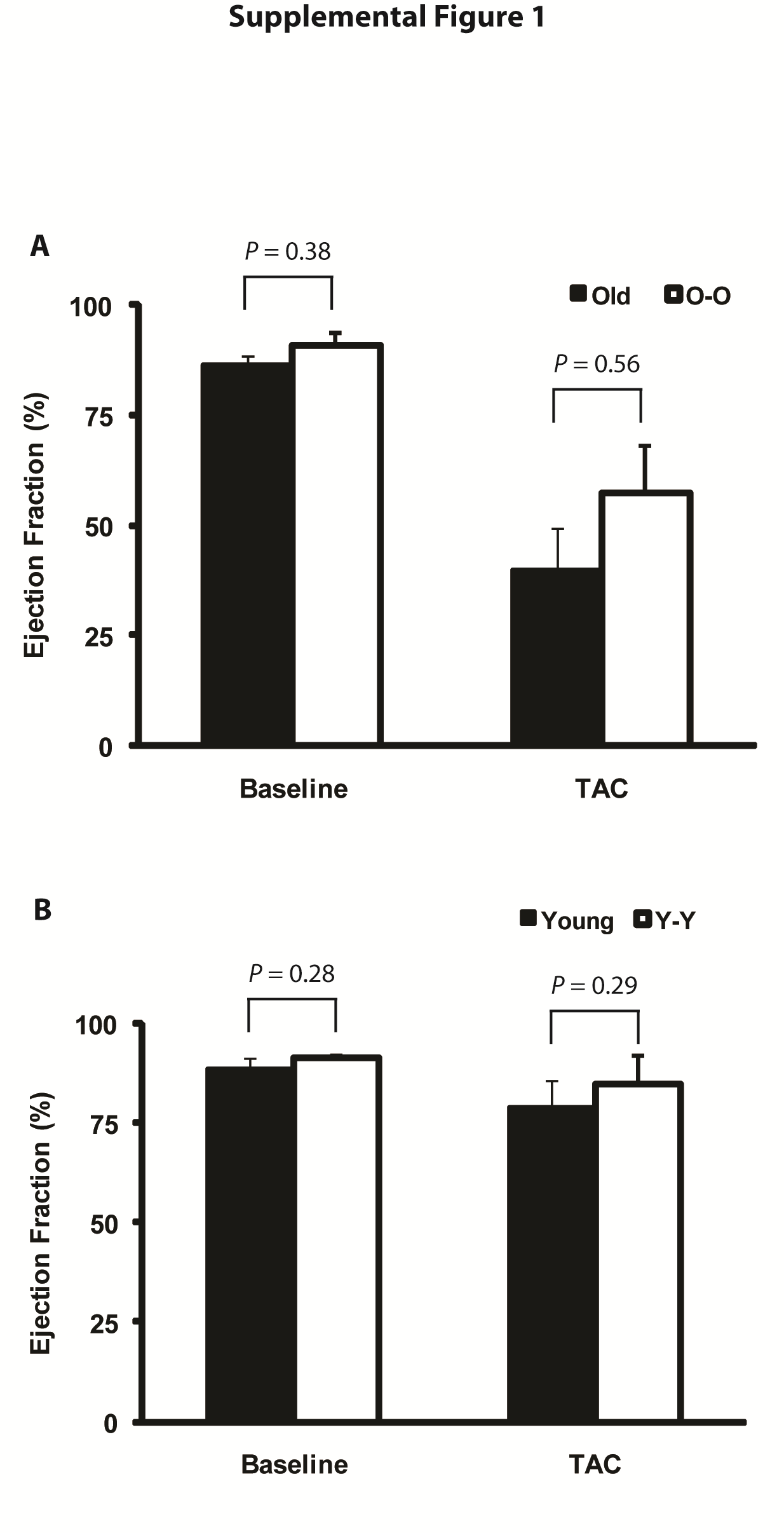

Supplement: Figure S1 — Bone marrow transplantation does not affect cardiac function at baseline prior to or after TAC in young mice bone marrow transplanted with young bone marrow (A) or old mice transplanted with old marrow (B) compared to their non-bone marrow transplanted surgical controls. n = 4-6 per group. (DOC) [file pone.0015187.s003.doc]
